# Exploring new rehabilitation pathways for stroke based on the comorbidity of post-stroke hypoesthesia with anxiety and depression

**DOI:** 10.7717/peerj.20679

**Published:** 2026-01-29

**Authors:** Yuyan Chen, Yusheng Zhao, Bangqi Wu, Yupei Cheng, Jingjie Huang, Chaoran Wang, Jing Bai, Yuxing Zhang

**Affiliations:** 1 First Teaching Hospital of Tianjin University of Traditional Chinese Medicine, Tianjin, China; 2 Tianjin University of Traditional Chinese Medicine, Tianjin, China

**Keywords:** Stroke, Hypoesthesia, Anxiety, Depression, Mechanisms, Predictive coding

## Abstract

**Background:**

Post-stroke hypoesthesia is a common yet often overlooked sequela, involving diminished capacities in touch, temperature, and pain perception. Recent studies suggest that sensory deficits not only hinder functional recovery but also show a high rate of comorbidity with anxiety and depression.

**Objective:**

This study aims to systematically integrate the neural mechanisms, perceptual processing features, and behavioral consequences of post-stroke hypoesthesia and emotional disorders, to explore their comorbid relationship and propose more targeted rehabilitation strategies based on these mechanisms.

**Methods:**

This review conducts an interdisciplinary literature search, integrating research from neuroimaging, cognitive neuroscience, and rehabilitation medicine, including 73 relevant studies. The keywords used in the screening are “Hypesthesia,” “Impaired Sensation,” “Anxiety,” and “Depression,” covering studies on sensory impairments and mood disorders. By comparing the sensory-emotion interaction mechanisms in stroke and non-stroke populations, a bidirectional model is constructed.

**Results:**

Findings indicate that post-stroke hypoesthesia results not only from structural damage in regions such as the thalamus, insula, and prefrontal cortex, but also from functional disruptions in perceptual processing. These impairments contribute to a closed-loop mechanism involving neural dysconnectivity and predictive coding dysfunction, which facilitates the emergence of anxiety and depression. In turn, these emotional disorders further suppress sensory recovery, significantly reducing patients’ motivation and rehabilitation compliance.

**Conclusion:**

Post-stroke hypoesthesia should be recognized as a critical etiological and maintaining factor in anxiety and depression. The coexistence of pathological and functional mechanisms underscores the need for rehabilitation strategies that transcend the boundaries of perception, emotion, and cognition. Developing a rehabilitation pathway centered on “sensory–emotional co-regulation” can facilitate early identification, subtype-specific intervention, and comprehensive support for emotional comorbidities following stroke.

## Introduction

As one of the early symptoms following stroke, hypoesthesia typically refers to a reduction in the intensity or clarity with which an individual perceives external stimuli—such as touch, pain, or temperature (Baier et al., 2014). It manifests as dullness, numbness, or a lack of responsiveness to stimulation, reflecting functional disruptions in sensory transmission pathways like the somatosensory and nociceptive systems (Baier et al., 2014). Although traditionally considered an accompanying symptom in neurological studies, recent clinical evidence suggests that stroke cases with hypoesthesia as the initial symptom exhibit distinct pathological and neuroimaging features (Akimoto et al., 2023). Therefore, post-stroke hypoesthesia warrants dedicated attention and systematic investigation.

Studies have shown that over half of stroke patients experience various forms of hypoesthesia during the acute or chronic phases, including deficits in light touch, fine tactile discrimination, and thermonociception (Gorst et al., 2019; Meyer et al., 2016). These impairments are especially common in regions such as the pharynx, oral cavity, soles of the feet, and limbs, often presenting as delayed perception of stimuli, difficulties in tactile recognition, or complete loss of sensation in affected areas (Meyer et al., 2016; Labeit et al., 2023; Schimmel et al., 2017; Parsons et al., 2016). Although these forms of sensory loss may not be as visibly evident as motor or speech impairments like hemiplegia or aphasia, they can severely impact a patient’s autonomy, ability to perform daily activities, and adherence to rehabilitation, thereby significantly reducing overall quality of life (Liu et al., 2022; Hoh & Semrau, 2025).

A substantial body of research has already confirmed the strong connection between post-stroke pain and emotional disorders (Murray, Bhanot & Bhargava, 2021). Mechanistic explanations include overlapping activation of pain and emotion regulation networks and the prolonged impact of chronic pain stimuli on the limbic system (Bastuji et al., 2016). However, in contrast, the emotional consequences of non-painful hypoesthesias—such as touch dullness or reduced thermal sensation—are often overlooked in clinical practice (Carey, Matyas & Baum, 2018). In reality, post-stroke hypoesthesia frequently co-occurs with a wide range of cognitive, emotional, and behavioral problems, particularly anxiety and depression (Price & Duman, 2020). Nevertheless, the neurobiological mechanisms underlying this comorbidity remain underexplored (Carey, Matyas & Baum, 2018).

Therefore, this review adopts an integrative perspective. It begins by examining foundational research beyond the stroke population to explore the bidirectional relationship and underlying mechanisms between hypoesthesia and anxiety/depression. It then delves into the neurological basis, behavioral consequences, and potential emotional triggers of sensory deficits in stroke survivors. Ultimately, the aim is to propose a cross-domain sensation–emotion interaction model that may offer new theoretical insights for understanding post-stroke comorbidity mechanisms and developing targeted intervention strategies.

## Survey methodology

This review focuses on the comorbidity mechanisms between post-stroke hypoesthesia and anxiety/depression, aiming to construct a sensory–emotion interaction model and explore targeted rehabilitation pathways. The literature synthesis is structured around three main areas: (1) the manifestations and mechanisms of hypoesthesia in emotional disorders among non-stroke populations; (2) the clinical spectrum and neural basis of post-stroke sensory deficits; (3) the interaction mechanisms between sensory and emotional disorders in stroke patients.

We systematically search PubMed, Web of Science, Cochrane Library, CNKI, Wanfang Data, VIP Database, and SinoMed for studies published from January 1, 2015, to May 31, 2025. The search strategy combines controlled vocabulary and free-text terms; core keywords include “hypesthesia,” “impaired sensation,” “anxiety,” and “depression.” Truncation and proximity operators are adapted to each database as appropriate. To minimize omissions, we also conduct backward citation tracking by hand-searching the reference lists of included studies. Inclusion criteria are: participants presenting both sensory impairment and mood disorder (anxiety and/or depression); publication between January 2015 and May 2025; and original empirical studies (including clinical/behavioral, fMRI/EEG, and neurophysiological research with observational or interventional designs). Exclusion criteria are: non-original articles such as case reports, letters to the editor, and editorials; studies that do not clearly link sensory impairment with anxiety and/or depression; and conference abstracts for which full methods and results are unavailable.

Two independent reviewers (Yuyan Chen and Yusheng Zhao) conduct study selection in two stages: an initial screening of titles/abstracts followed by full-text assessment of potentially eligible records. Discrepancies are resolved through discussion to reach consensus; when consensus cannot be achieved, the third reviewer (Yupei Cheng) adjudicates the final decision. To ensure methodological rigor and topical relevance, we combine multi-database searching with backward citation tracing to enhance coverage; at inclusion we evaluate whether study designs are clearly described, whether sensory and mood measures use validated instruments or diagnostic criteria, and whether sample sizes are commensurate with the study design. Data extraction covers participant characteristics, study design and setting, sensory phenotypes (*e.g*., hyporesponsiveness/low registration and focal deficits), emotional outcomes (anxiety/depression scales or diagnoses), neural correlates (connectivity alterations and key nodes such as the thalamus, insula, and prefrontal cortex), and rehabilitation-relevant findings. Evidence synthesis integrates perspectives from neuroimaging, cognitive neuroscience, and predictive coding theory, and employs the Research Domain Criteria (RDoC) framework to elucidate the mediating role of sensory processing in emotional dysfunction. On this basis, we propose a bidirectional sensory–emotion model and provide theoretical support for mechanism-based stroke rehabilitation.

**Figure 1:**
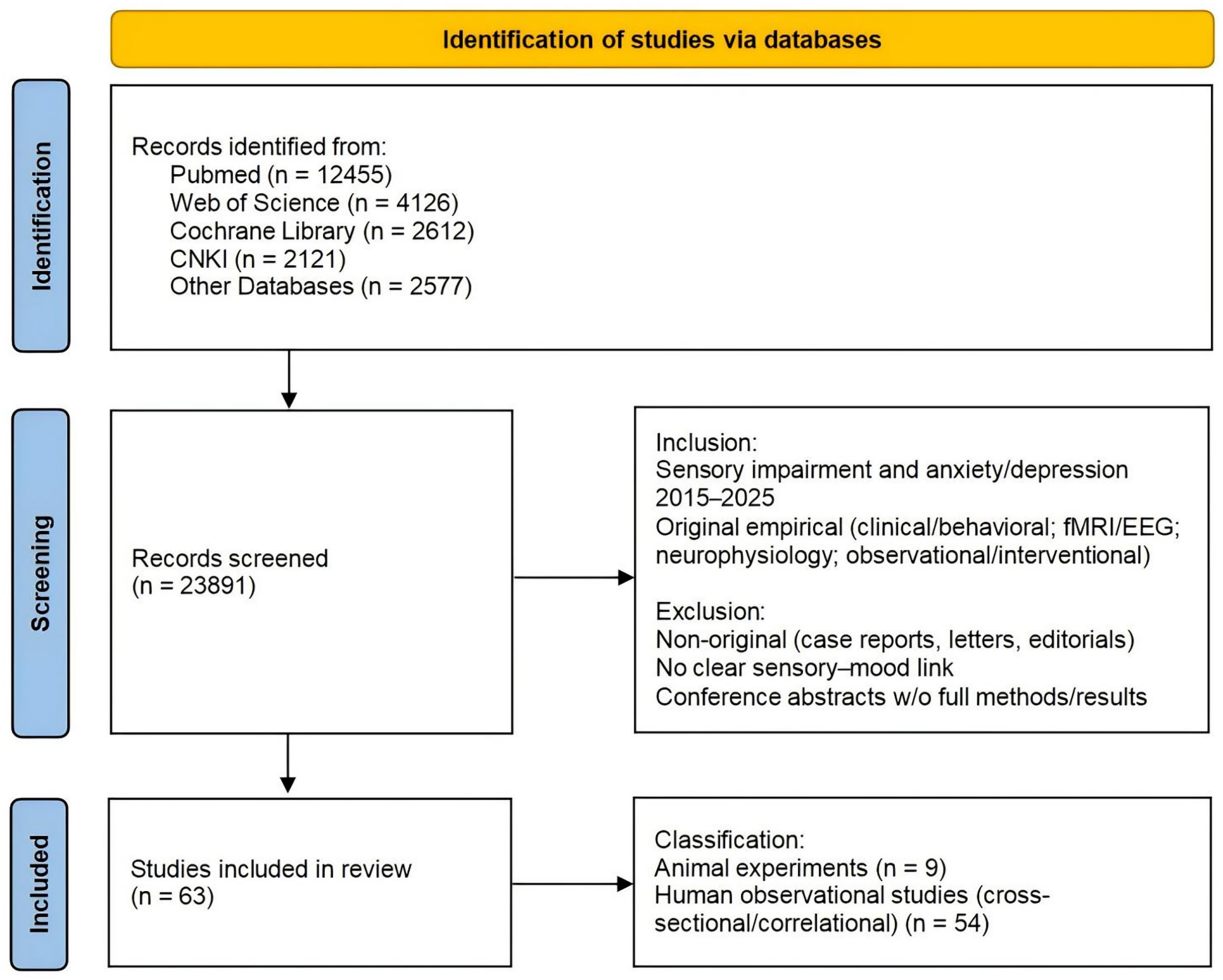
Flow of literature identification, screening, and inclusion for the review. This flow diagram illustrates the identification and screening process of studies included in the review. Records were retrieved from multiple databases (PubMed, Web of Science, Cochrane Library, CNKI, and others), screened according to predefined inclusion and exclusion criteria, and finally classified into animal experiments and human observational studies.

The search initially identifies 23,891 records; after title/abstract screening and full-text review, 63 studies are included in the qualitative synthesis. The complete screening process, numbers excluded at each stage with reasons, and the final study set are presented in the Fig. 1.

## The relationship between hypoesthesia and anxiety

### Specific manifestations of hypoesthesia in individuals with anxiety

#### Low-registration type hypoesthesia

Low sensory registration refers to a reduced responsiveness to external stimuli, characterized by elevated sensory thresholds and delayed reactions to physical inputs such as light touch, temperature changes, or low-intensity pain (Neufeld et al., 2021). Patients often report “not feeling the cold,” “only realizing I was touched after the fact,” or that “my body feels numb,” and may show emotional detachment, difficulty recalling bodily sensations, or vague expression during clinical interviews (Itahashi et al., 2020).

Although anxiety has traditionally been associated with sensory “hypersensitivity,” recent studies indicate that individuals with anxiety may also exhibit low-registration hypoesthesias (Itahashi et al., 2020; Aaron et al., 2025). Favaretto et al. (2022) noted that pain sensitivity is significantly modulated by affective temperament, suggesting high heterogeneity between anxiety states and sensory processing. Hammud et al. (2025) found that adolescents with anxiety showed delayed responses to stimuli such as temperature, touch, and mild pain, accompanied by elevated neural arousal thresholds and reduced electrodermal activity (EDA) and heart rate variability.

Even in healthy individuals without psychiatric diagnoses, higher anxiety levels are significantly associated with reduced tactile sensitivity. Occlusal tactile acuity tests have shown that anxious individuals have difficulty distinguishing fine differences in thickness, a trait negatively correlated with pain catastrophizing (Coppola et al., 2024). Ranford et al. (2020) reported that patients with functional neurological disorder (FND) and a history of anxiety more often show dulled touch/temperature responses, suggesting low registration as a vulnerability factor for anxiety-related FND. This phenomenon is also observed in children with autism spectrum disorder (ASD), where sensory under-responsiveness is strongly associated with increased intolerance of uncertainty and anxiety, often resulting in repetitive behaviors (Wigham et al., 2015). Patients with pathological illness anxiety (PIA), though often perceived as overly focused on bodily sensations, have been shown in experimental studies to exhibit reduced tactile perception (Wolters et al., 2023). Wolters et al. (2023), using the Somatic Signal Detection Task (SSDT), found that PIA patients had impaired recognition of faint tactile stimuli when emotionally charged words were presented, indicating that semantic anxiety load and attentional resource depletion can induce functional sensory suppression. Low registration has emerged as a core perceptual phenotype of anxiety across various populations, suggesting it may play a critical role in both the development and vulnerability of anxiety disorders.

#### Regional hypoesthesia

In addition to global sensory deficits, individuals with anxiety often show regional hypoesthesias. Livianos et al. (2016) found that 65.59% of anxiety patients exhibited significant asymmetrical tactile hypoesthesia at the medial and lateral malleolus. This sensory dullness does not stem from peripheral nerve damage but likely reflects disruptions at the thalamic level in the central sensory–cognitive–emotional regulation pathway. Such localized hypoesthesia is regarded as a “neurological soft sign,” indicating that anxiety disorders may initially manifest as non-anatomical abnormalities in sensory and cognitive functions (Livianos et al., 2016). This finding provides a novel neurobiological basis for the identification and systematic assessment of anxiety-related symptoms.

### A bidirectional reinforcement system between hypoesthesia and anxiety

Recent research views hypoesthesia not as a byproduct of anxiety but as an active contributor to its development (Wigham et al., 2015). Contrary to the traditional one-way model that characterizes anxiety as a state of heightened sensory reactivity, growing evidence suggests that certain anxious individuals actually show reduced sensitivity to external stimuli—particularly in bodily channels like touch and pain. This diminished sensory input undermines immediate environmental responsiveness and disrupts the individual’s internal “safety” system, forcing threat prediction and emotional mobilization processes into an overloaded state, which in turn exacerbates anxiety. Thus, hypoesthesia and anxiety form a reciprocal dynamic loop that drives the onset, persistence, and intensification of anxiety.

Wigham et al. (2015) found that hypoesthesia heightens intolerance of uncertainty, increasing anxiety and repetitive, stereotyped behaviors (RRBs). In this feedback loop of sensory loss → anxiety amplification through uncertainty → behavioral rigidity, individuals attempt to compensate for disrupted perceptual continuity through rigid behavioral control (Wigham et al., 2015). This indicates that hypoesthesia may not only be a result of anxiety but also a foundational trigger in the emergence of pathological behavioral patterns. Livianos et al. (2016) demonstrated that patients with generalized anxiety disorder (GAD) or mixed anxiety-depression exhibited asymmetric tactile hypoesthesia around the ankles, and those with sensory deficits scored significantly higher on anxiety measures. Impaired tactile input compromises real-time awareness of bodily states, weakening the internal verification of safety—“Am I safe right now?”—and forcing reliance on cognitive hypotheses and semantic inference to interpret ambiguous bodily signals (MacLeod & Mathews, 2012). This increases the likelihood of predictive threat bias, a core feature of anxiety (MacLeod & Mathews, 2012). Essentially, this represents the breakdown of the “sensory confirmation system,” trapping individuals in a cycle of uncertainty and anticipatory fear (Elhamiasl et al., 2022).

In studies of healthy populations, Coppola et al. (2024) found that individuals with higher anxiety levels and catastrophizing pain beliefs performed poorly on occlusal tactile acuity (OTA) tests. Wolters et al. (2023) further showed that individuals with PIA demonstrated significantly reduced tactile recognition under semantic interference related to illness, indicating that emotionally loaded language can deplete attentional resources and lead to functional sensory suppression. Unlike structural-damage deficits, this inhibition is functional, arising from resource competition within the emotion–attention system (Park & Moghaddam, 2017). It reveals how anxiety can actively disrupt the brain’s capacity to regulate sensory processing (Vytal et al., 2012).

Ultimately, the relationship between hypoesthesia and anxiety is not a simple linear causality but a multidirectional interaction across cognitive regulation, emotional activation, and behavioral adaptation. Diminished sensory input reduces perceived environmental stability, triggering anxiety and hyperarousal. In turn, anxiety feeds back to suppress sensory processing through attentional overload and emotional interference—creating a self-reinforcing cycle of hypoesthesia → anxiety → further hypoesthesia.

## The relationship between hypoesthesia and depression

### Specific manifestations of hypoesthesia in individuals with depression

#### Low-registration type hypoesthesia

An increasing body of research indicates a strong link between sensory processing patterns and depressive symptoms. Engel-Yeger et al. (2018), in a study of outpatients with major affective disorders, found that low sensory registration significantly predicted higher levels of self-reported depression and hopelessness, and acted as a mediator linking irritability/risk-taking tendencies to the development of depressive symptoms. Additionally, research by Bitsika, Sharpley & Mills (2016) revealed that among adolescents with ASD, low registration was one of the strongest predictors of depressive symptoms—surpassing even core diagnostic features like social communication deficits.

#### Regional hypoesthesia

Patients with major depressive disorder (MDD) not only report subjective numbness but also exhibit measurable localized sensory abnormalities. Using a Wartenberg pinwheel, Livianos et al. (2015) found that individuals with depression had a significantly higher rate of asymmetrical tactile hypoesthesia at the inner and outer ankle, categorizing this as a form of “neurological soft sign”. Crucianelli, Radziun & Ehrsson (2024), through a dynamic thermal matching task, found that individuals with higher levels of depression showed significant impairments in detecting temperature changes on the palm, indicating that depressive states are associated with sensory dysfunction even at the level of the skin—particularly in temperature perception, which is closely linked to emotional processing.

#### Multisensory decline

With aging, sensory function declines, harming quality of life and closely linking to mood disorders, especially depression. Unlike developmental deficits in youth, age-related loss reflects regressive sensory deprivation with broader, longer-lasting effects on emotion and social functioning. In large population samples, Liljas et al. (2020) and Sacknovitz et al. (2024) found a significant linear relation between the number of sensory deficits (vision, hearing, touch) and depression prevalence—the more impairments, the higher the risk. Reduced tactile sensitivity may also diminish awareness of physical discomfort, potentially leading to health-related anxiety. Sempere-Bigorra et al. (2023) compared diabetic and non-diabetic older adults and found that dulled temperature and vibration perception were more common in depressed elderly individuals with diabetes, and that the severity of these deficits correlated significantly with depressive symptoms and reduced quality of life.

#### Modality-dependent and right-lateralized sensory bias

Extensive research confirms that depressive disorders are accompanied by widespread abnormalities in pain regulation. Meta-analyses by Thompson et al. (2016) and Scaini et al. (2025) indicated that individuals with depression exhibit generally elevated pain thresholds, especially in response to low-intensity thermal or electrical pain stimuli. However, this dullness is not uniformly distributed across the body. Bär et al. (2005) found that MDD patients exhibited increased tolerance to thermal and electrical pain on the right side of the body, but showed hypersensitivity to ischemic muscle pain. This pattern of modality dependence combined with right-sided bias may reflect imbalances in lateralized regulation between the limbic system and prefrontal cortex—particularly decreased excitability in the right insular cortex, potentially leading to impaired recognition, integration, and response to pain and emotional signals (Bär et al., 2005).

### A bidirectional reinforcement system between hypoesthesia and depression

The comorbidity between hypoesthesia and depression is closely tied to sensory input, cognitive processing, and emotional regulation. Current findings suggest that hypoesthesia includes both pathological sensory deficits—resulting from neural structural damage—and functional sensory disruptions, which stem from prediction errors and suppression within the emotional system. These layered dysfunctions form an integrated model of perceptual–emotional dysregulation.

In cognitive neuroscience, the predictive coding framework has become a key theory for understanding emotional disorders (Sheffield et al., 2024). It posits that the brain does not passively receive stimuli but actively predicts incoming information based on internal models, adjusting perception and response through the comparison of prediction and actual input (Sheffield et al., 2024; Aitchison & Lengyel, 2017). Gilbert, Wusinich & Zarate (2022) noted that depressed individuals struggle to generate accurate predictions or update internal models, leading to persistent prediction errors. In depressive states, sensitivity to environmental stimuli becomes distorted—often manifesting as a blunted response to positive inputs, including tactile and thermal sensations (Gotlib & Joormann, 2010). This “signal modulation failure” disrupts perceptual efficiency and undermines the coherent sensing of bodily and emotional states, resulting in a persistent disconnect between feeling and emotion (Schifferstein & Desmet, 2007).

This hypothesis was further validated by Crucianelli, Radziun & Ehrsson (2024), who found that individuals with higher levels of depression performed poorly on palm thermal perception tasks, failing to detect temperature changes. The dysfunction of the predictive coding system offers a novel lens to understand hypoesthesia in depression: the misalignment between perception and prediction not only dulls responses to real stimuli but also erodes one’s stable sense of self, intensifying negative affect and perpetuating a dysfunctional cycle of cognition, emotion, and perception (Ubl et al., 2015). Furthermore, hypoesthesia may reduce the ability to detect positive environmental cues, weakening activation of emotional regulation mechanisms. It also limits awareness of discomfort, risks, and emotional signals, disrupting internal and external feedback systems (de Cruys & Van Dessel, 2021). Thus, hypoesthesia is not only a symptom of depression but also a crucial mediator within its perceptual–emotional dysfunction.

Engel-Yeger et al. (2018) found that low sensory registration significantly predicted higher levels of self-reported depression and hopelessness. Notably, their research showed that low registration mediates the transition from irritability/risk-taking tendencies (often seen in hypomania) to depressive states—suggesting that hypoesthesia not only reflects depressive symptoms but may amplify emotional deterioration. Reduced sensitivity to positive stimuli (*e.g*., touch, sunlight) deprives the emotional system of sensory inputs necessary for positive affect regulation (Engel-Yeger et al., 2018). Simultaneously, it limits detection of threats or discomfort, trapping individuals in a cycle of numbness, helplessness, and hopelessness—further fueling cognitive inhibition, emotional blunting, and motivational decline. In another study, Engel-Yeger et al. (2016) found that low registration often coexists with high sensitivity/avoidance traits, making it difficult for depressed individuals to adapt to daily stimuli. They may fail to perceive calming signals while overreacting to minor changes. This imbalance in sensory regulation undermines emotional stability and adaptive functioning (Engel-Yeger et al., 2016). Paquet et al. (2022) noted that if low registration is paired with reduced sensory-seeking behavior, individuals may be unable to actively acquire positive stimulation or internally activate emotional responses—resulting in a “sensory deprivation–feedback paralysis–emotional stagnation” cycle. In such states, even when the environment changes, adaptive responses may not emerge, leaving the individual in a state of “static collapse” or “latent breakdown” (Paquet et al., 2022).

During childhood and adolescence, when the sensory system is still maturing, its developmental quality is critical for emotional and psychological regulation. Bitsika, Sharpley & Mills (2016) found that adolescents with ASD who showed sensory under-responsiveness struggled to identify or name emotional states in response to ambiguous, stressful, or novel stimuli. Without adequate somatic cues, they often resorted to maladaptive emotional strategies such as avoidance, defense, or withdrawal. This disconnection between sensation and emotion may predispose individuals to chronic stress vulnerability, laying a foundation for future depression (Bitsika, Sharpley & Mills, 2016).

In the elderly, this mechanism manifests as a progression from sensory decline to emotional withdrawal. Sacknovitz et al. (2024) found a significant positive correlation between multihypoesthesia (vision, hearing, touch) and depression rates in older adults. Reduced sensory input narrows the channels through which individuals engage with the world, breaking both external interaction and internal bodily anchoring—leading to a loss of connection and control (Sacknovitz et al., 2024). Sempere-Bigorra et al. (2023) further found that elderly patients with diabetes displayed more severe deficits in temperature and vibration perception, which impaired health awareness and care behaviors, increasing helplessness and emotional exhaustion. Liljas et al. (2020) emphasized that multihypoesthesia disrupts social and self-care systems in older adults, contributing to functional isolation and cognitive decline, ultimately undermining both quality of life and psychological resilience.

In summary, depression and hypoesthesia form a bidirectional reinforcement rather than a one-way cause–effect link. Hypoesthesia weakens perception of self and world; depression, through emotional suppression and loss of motivation, further reduces engagement with external stimuli. This loop—sensory decline → depression → further decline—appears across ages and diagnoses, underscoring the foundational role of sensory and cognitive systems in emotional health and the need for greater clinical attention to their dysregulation.

## Exploring the relationship between post-stroke hypoesthesia and anxiety/depression

### Clinical spectrum and impacts of post-stroke hypoesthesia

Post-stroke hypoesthesia extends beyond diminished limb touch perception; it encompasses a wide range of multisystem, cross-regional sensory pathway disruptions, including the pharynx, oral cavity, soles of the feet, face, and spatial perception (Akimoto et al., 2023; Meyer et al., 2016; Labeit et al., 2023; Schimmel et al., 2017; Parsons et al., 2016; Di Gregorio et al., 2023). These disturbances exhibit a clinical profile characterized by “subtype differentiation with mechanistic convergence,” warranting independent attention in both neurorehabilitation and emotional interventions.

Pharyngeal sensory loss manifests as difficulty in detecting objects within the oral cavity—such as saliva—and is a major risk factor for aspiration and pneumonia following stroke, representing a distinct sensory pathway injury (Labeit et al., 2023). Oral tactile impairment involves elevated thresholds in the tongue, cheeks, and lips, leading to difficulties in locating food within the mouth. Patients often subjectively report “I can’t feel the food” or “I can’t chew, but it’s not my teeth” (Schimmel et al., 2017). Tactile impairment in the upper and lower limbs is the most commonly reported form, including numbness, elevated two-point discrimination thresholds, vague temperature perception, and loss of proprioception, particularly pronounced in the affected-side fingers and soles (Meyer et al., 2016; Parsons et al., 2016). Contralesional spatial neglect and fine touch deficits—often resulting from right parietal-insular network damage—are core symptoms involving sensory avoidance and attentional withdrawal from one side of space (Di Gregorio et al., 2023). These are mechanistically consistent with regional hypoesthesias. Despite the diversity in clinical presentations, all subtypes share a common feature: structural disruption of sensory input pathways → reduced perceptual certainty, which lays the biological foundation for increased emotional uncertainty.

Hypoesthesia affects not only physical function but also emotions, motivation, and lifestyle. Parsons et al. (2016) showed that post-stroke plantar sensory loss triggers postural compensation and coordination disruption, reducing confidence and willingness to retrain and increasing distress and functional withdrawal. Carlsson, Gard & Brogårdh (2018), through qualitative interviews, found that stroke survivors with upper-limb sensory deficits commonly reported that “my hand doesn’t feel like part of my body” or “I can’t grasp with confidence,” leading to spontaneous disuse of the affected side. This “learned non-use” further entrenches body schema disruptions and functional regression. Carey, Matyas & Baum (2018) noted that even without obvious motor deficits, sensory loss alone markedly reduces daily participation and is linked to avoidant social behavior, heightened anxiety, and depressive tendencies.

### Neural mechanisms of post-stroke hypoesthesia

The pathological basis of post-stroke hypoesthesia is not simply a weakening of sensory input signals but rather a systemic disruption of integration and preparatory mechanisms. This involves not only direct damage to the thalamo-cortical pathways but also functional degradation of higher-order integration centers and widespread disconnection of interregional neural networks. The following summarizes these mechanisms from three key perspectives.

#### Direct damage and conduction interruption in the thalamo-cortical pathways

Stroke often affects the thalamus and its ascending projection pathways, especially the superior thalamocortical radiation (STR)—an essential relay for multimodal sensory information (*e.g*., touch, pain, proprioception) to reach the primary somatosensory cortex (S1) (Meyer et al., 2016). Meyer et al. (2016) found that lesions in the STR region were strongly associated with deficits across multiple sensory modalities following stroke. Beyond direct projection damage, stroke may also lead to white matter abnormalities remote from the lesion site. Koh et al. (2021) noted that even when S1 remains structurally intact, microstructural degradation can occur in remote tracts such as the corpus callosum, thalamo-cortical fibers, and pontocerebellar tracts. This phenomenon, referred to as “structural connectional diaschisis”, explains why some stroke patients show significant sensory loss despite no clear focal damage in primary sensory regions (Koh et al., 2021).

#### Functional degradation of higher-order integration pathways and perceptual disturbances

Beyond primary sensory input, tactile and somatosensory information must be integrated, interpreted, and made conscious through higher-order cortical areas (Preusser et al., 2015). Preusser et al. (2015) proposed that the insula, operculum, putamen, and prefrontal cortex together form a “ventral somatosensory pathway,” a crucial network for generating sensory awareness. Even when S1 is preserved, damage to this pathway can cause pronounced tactile loss and awareness deficits (Preusser et al., 2015). Rullmann, Preusser & Pleger (2019) further pointed out that sensory awareness results from multilevel neural processing, including primary cortical signal recognition, posterior parietal spatial analysis, and prefrontal attentional allocation. Damage to any of these nodes may lead to blurred perceptual experiences, such as “knowing I was touched but not sensing the details” (Rullmann, Preusser & Pleger, 2019).

The integration of pharyngeal and oral sensory signals is also particularly vulnerable to stroke. Cabib et al. (2017), using evoked potential studies, found significant delays in cortical activation following pharyngeal stimulation, especially in the stroke-affected hemisphere. Schimmel et al. (2017) observed significantly elevated tactile thresholds in the tongue and lips of stroke patients, suggesting bilateral impairment of the trigeminal nerve and associated central pathways.

#### Disintegration of functional network connectivity and interhemispheric imbalance

Recent resting-state functional magnetic resonance imaging (fMRI) studies have shown that stroke disrupts not only localized regions but also large-scale functional connectivity across neural networks. Goodin et al. (2018) found reduced connectivity between primary and secondary somatosensory cortices in stroke patients, with interhemispheric coordination particularly impaired. Left hemisphere lesions mainly reduced bilateral coordination efficiency, while right hemisphere lesions more often activated compensatory right frontoparietal networks, manifesting in altered somatosensory perception (Goodin et al., 2018).

Stroke also impacts “early sensory processing”. de Haan, Stoll & Karnath (2015) demonstrated that even right hemisphere stroke patients without clinical spatial neglect showed reduced reactivity to stimuli on the left side, occurring prior to conscious awareness. This suggests that sensory degradation may begin at the level of the thalamus or primary sensory cortices. Moreover, Di Gregorio et al. (2023), using EEG combined with structural equation modeling (SEM), revealed for the first time a layered disruption in sensory processing from pre-stimulus to behavioral response in patients with left hemineglect (LHN). They found that perceptual biases could appear during early neural processing—even in the absence of overt behavioral deficits—implying potential subclinical sensory asymmetry (Di Gregorio et al., 2023). This suggests that when the brain remains in a state of persistent prediction-feedback mismatch, it can generate an internal experience of “unreliable perception,” which may trigger emotional defenses and avoidance behaviors.

### Exploring the relationship between post-stroke hypoesthesia and anxiety/depression

#### Functional brain region associations between post-stroke hypoesthesia and emotional disorders

Post-stroke hypoesthesia is not merely a localized interruption in sensory input pathways—it also involves functional disintegration among central brain regions closely associated with emotional regulation (Zhang, Deng & Xiao, 2024; Fox, 2018). Recent studies have shown that dysfunction in areas such as the insula, prefrontal cortex, and hippocampus plays a central role in the comorbidity of sensory deficits with anxiety and depression after stroke (Price & Duman, 2020; Zhang, Deng & Xiao, 2024; Shi et al., 2023; Timbie & Barbas, 2015). This network-level disconnect manifests as decreased sensory capacity, disrupted interpretation of cognitive signals, and hyperactivation of negative emotional circuits—together forming a pathological loop of “sensory loss → emotional amplification.”

**(1) Insular cortex: A central hub bridging sensation and emotion**

The insula is a key integrative center for internal and external sensory information, acting as a bridge between somatosensory cortices (S1/S2) and the limbic system (*e.g*., amygdala, prefrontal cortex) (Zhang, Deng & Xiao, 2024). Its posterior region primarily processes basic sensory inputs such as temperature, pain, touch, and proprioception, while the anterior insula is involved in the subjective awareness of bodily states, emotional valuation, and the generation of anxiety- and depression-related drives (Wiech et al., 2014; Sliz & Hayley, 2012; Jones, Ward & Critchley, 2010; Lu et al., 2016). Stroke-related insular dysfunction can impair sensory processing and disrupt its connectivity with regions like the prefrontal cortex and thalamus (Zhang, Deng & Xiao, 2024). This may result in either heightened emotional reactivity to bodily signals or emotional numbness—both of which can intensify anxiety responses and increase vulnerability to depression (Jones, Ward & Critchley, 2010; Terasawa et al., 2015).

**(2) Hippocampus: A regulator of sensory memory and emotion**

The hippocampus is traditionally recognized for its role in memory processing, but recent research highlights its involvement in emotional regulation—especially its ventral portion (vHPC), which is highly sensitive to anxiety modulation (Chen et al., 2022; Ramos & Morón, 2022). The hippocampus, through its circuits with the amygdala and prefrontal cortex, helps regulate stress responses and emotional salience (Shi et al., 2023; Adhikari et al., 2015). Dysregulation in this circuitry has been strongly linked to various anxiety disorders. If stroke affects the thalamo-hippocampal pathway, patients may exhibit a paradoxical state of amplified anxiety alongside suppressed interoceptive perception—contributing to a vicious cycle of “sensory absence → emotional escalation” (Scharf et al., 2023). Additionally, the hippocampus plays a pivotal role in the encoding and retention of negative information in depression (Chaudhary et al., 2022). Depressed individuals often experience recurring negative memories, which are associated with weakened functional connectivity between the hippocampus and prefrontal cortex (Moda-Sava et al., 2019; Cha et al., 2016). Damage to these pathways after stroke may cause individuals to recall and evaluate residual sensory experiences with a persistent negative bias, leading to emotional conflicts such as “I don’t feel anything, but it still feels unbearable” (Cha et al., 2016). Structural imaging studies have further demonstrated that reduced hippocampal volume is positively correlated with the risk of post-stroke depression, providing anatomical evidence of its central role in the interplay between emotion, memory, and sensory processing (Spellman & Liston, 2020; Liston et al., 2014; Oathes et al., 2015).

**(3) Prefrontal cortex: A higher-order hub for sensory valuation and emotional control**

The prefrontal cortex (PFC) serves as a central integration zone for sensory selection, meaning attribution, and emotional regulation (Barbas, Zikopoulos & Timbie, 2011). Although it may not be directly damaged in stroke, its functional connectivity with the sensory system is often disrupted due to remote decoupling (Krain et al., 2008; Ferenczi et al., 2016). The dorsolateral prefrontal cortex (dlPFC) plays a key role in perception-attention-goal selection, while the orbitofrontal cortex (OFC) governs the attribution of emotional value (Resstel, Souza & Guimarães, 2008). Following stroke, these regions often show disordered selective attention mechanisms, heightened vigilance toward ambiguous stimuli, and a tendency to negatively interpret sensory signals—resulting in a “sensory reduction → overinterpretation” pattern that amplifies anxiety (Spellman & Liston, 2020; García-Cabezas & Barbas, 2017). Structural MRI and fMRI studies also reveal that in depression, dlPFC shows decreased activation during emotion regulation and sensory processing tasks, while the OFC exhibits strengthened connectivity with limbic structures like the insula and hippocampus, reflecting a heightened focus on negative information (Bagot et al., 2015; Kirkby et al., 2018). Moreover, functional connectivity studies have shown significant weakening in the connections between the S1/S2 regions and the PFC after stroke—particularly in patients with comorbid depression—indicating that deficits in sensory processing and emotional cognitive regulation may co-occur (Goodin et al., 2018).

**(4) Global network disintegration and functional coordination imbalance**

Damage to the thalamo-cortical pathways caused by stroke not only disrupts external sensory input but may also impair cross-regional communication among the prefrontal cortex, insula, and hippocampus (Kirkby et al., 2018; van der Horn et al., 2017). Resting-state fMRI shows reduced functional connectivity among the prefrontal cortex, hippocampus, and insula in stroke; these same links are dysfunctional in non-stroke anxiety disorders (Goodin et al., 2018; Bijsterbosch et al., 2014; Nicolas et al., 2023). This overlap points to shared network-level abnormalities, with impaired cross-regional reappraisal capacity fueling the vicious interplay between sensory deficits and anxiety.

Neuroimaging research indicates that widespread disruption of the PFC–hippocampus–insula–thalamus network is a key mechanism underlying the comorbidity of hypoesthesia and depression after stroke (Price & Duman, 2020; Siegle et al., 2007). The integrative function of the prefrontal cortex in assigning emotional significance to sensory input diminishes, making it difficult for individuals to process ambiguous signals positively (Moda-Sava et al., 2019; Yan et al., 2018). At the same time, the insula’s role in monitoring internal bodily states is impaired due to network decoupling, weakening subjective emotional recognition and regulation (Zhang, Deng & Xiao, 2024; Manes, Paradiso & Robinson, 1999; Clark et al., 2008). The hippocampus, as a central integrator of emotion, memory, and perception, shows volume reduction and decreased connectivity after stroke, enhancing its bias toward negative information and reinforcing the “hypoesthesia → negative appraisal → depression” pathway (McEwen, Nasca & Gray, 2016). Damage to white matter tracts and thalamic relay segments caused by stroke is a major contributor to the breakdown of this integrative network (Zhang et al., 2021). This multi-region coordination imbalance leads to a “perceptual disconnection” that makes stroke survivors particularly vulnerable to feelings of helplessness and deepening depression during the recovery process.

Ultimately, post-stroke anxiety and depression reflect not only dysfunction within emotional centers but a rupture in the interaction between sensory processing and emotional regulation. Disrupted interregional connectivity reduces the brain’s capacity to reconstruct meaning from external stimuli, trapping patients in “sensory loss plus emotional instability.” This mechanism underscores the need for interventions that address mood symptoms while rebuilding the sensory–cognitive–emotional network through a systems-level approach.

#### Mechanisms of post-stroke hypoesthesia and comorbid anxiety/depression

**(1) The amplifying interaction between post-stroke hypoesthesia and anxiety**

Post-stroke hypoesthesia is not a singular type of perceptual disorder, but rather a hybrid condition involving both pathological and functional mechanisms. On one hand, direct damage to key brain regions such as the thalamus and the primary/secondary somatosensory cortices (S1/S2) leads to pathological loss of pain, touch, and temperature pathways, thereby weakening the individual’s ability to promptly detect environmental safety or threat signals (Rullmann, Preusser & Pleger, 2019; Lutkenhoff et al., 2015). On the other hand, under the influence of cross-regional functional imbalance among cognitive and emotional centers—such as the prefrontal cortex, insula, and hippocampus—patients may develop biased sensory processing, altered emotional thresholds, and abnormal attentional allocation, manifesting as functional hypoesthesia (Price & Duman, 2020; Wolters et al., 2023; Kirkby et al., 2018; van der Horn et al., 2017; Siegle et al., 2007).

This hybrid form of sensory loss becomes a major trigger, maintaining factor, and aggravating mechanism for anxiety symptoms in stroke patients. The aspect severs the internal “confirmation pathway” by which individuals assess their physical state, triggering a persistent sense of uncertainty and loss of control. This, in turn, heightens vigilance to potential threats and promotes catastrophic thinking—key drivers of anxiety. Livianos et al. (2016, 2015) observed significant localized tactile impairments in patients with generalized anxiety disorder (GAD) and anxiety–depression comorbidity, suggesting a “soft neurophenotype” that may also serve as a prodromal marker in stroke survivors.

Meanwhile, anxiety reinforces hypoesthesia, creating a vicious feedback loop dominated by functional mechanisms. Persistent anxiety leads to sympathetic overactivation and parasympathetic suppression, resulting in “physiological dampening”—a defensive “turn down the volume” response that temporarily reduces emotional overload but also cuts off pathways to bodily feedback and safety perception (Viljoen, Claassen & Mare, 2013; Giuliano et al., 2017; Kenwood, Kalin & Barbas, 2022).

Furthermore, anxiety creates semantic-attentional competition with the sensory system. Wolters et al. (2023) found that individuals with illness anxiety showed reduced tactile detection under emotionally loaded semantic cues (*e.g*., “illness”), indicating that emotional content can directly suppress sensory processing. Stroke patients are particularly vulnerable during early rehabilitation, experiencing “numbness–panic” episodes due to both impaired somatic feedback and heightened loss of control (Wigham et al., 2015). Favaretto et al. (2022) also found in non-clinical populations that higher anxiety levels are associated with lower pain and tactile sensitivity, suggesting that functional sensory dampening may be widespread in anxiety-prone individuals. In stroke contexts, this bidirectional influence results in a self-reinforcing cycle: “hypoesthesia → anxiety activation → further sensory suppression.”

**(2) The amplifying interaction between post-stroke hypoesthesia and depression**

Post-stroke sensory system degradation may initiate, sustain, and exacerbate depressive disorders through a combination of pathological and cognitive-emotional pathways. This manifests as a downward cascade of “sensory blunting → emotional numbness → motivational inhibition”. Engel-Yeger et al. (2018) identified low sensory registration as a key predictor of hopelessness and motivational decline in individuals with major depressive disorder. Stroke patients often experience “affected-side numbness” and a sense of “somatic disconnection,” making it difficult to respond to environmental stimuli or generate emotional engagement—deepening their sense of withdrawal from the world (Parsons et al., 2016). Sacknovitz et al. (2024) reported a strong linear correlation between multihypoesthesia and depression severity. Stroke further compresses already declining sensory pathways, leaving individuals in a dual state of isolation, “I can’t feel the world, and the world doesn’t feel me”, thus eroding both their subjective sense of presence and self-efficacy (Sacknovitz et al., 2024).

In depressive states, individuals often exhibit predictive processing deficits, leading to a breakdown in feedback between the perceptual and emotional systems. Gilbert, Wusinich & Zarate’s (2022) predictive coding model posits that depressed individuals struggle to update internal models in response to external stimuli, resulting in ignored or misread sensory signals. Crucianelli, Radziun & Ehrsson (2024) found that depressed individuals had significant difficulties recognizing temperature changes during thermal matching tasks. These patients frequently report cognitive dissonance such as “I know I should feel something, but I just don’t,” reflecting a failure to generate positive affect or initiate adaptive emotional regulation (Crucianelli, Radziun & Ehrsson, 2024). After stroke, this mechanism is further intensified due to structural and functional neural damage: disruptions in pathways such as the thalamus and S1/S2 significantly weaken primary sensory input, while impaired integrative processing among cognitive-emotional centers—including the insula, prefrontal cortex, and hippocampus—leads to the accumulation of persistent prediction errors and a prolonged disconnection between sensory and emotional information pathways (Rullmann, Preusser & Pleger, 2019; Zhang, Deng & Xiao, 2024; Spellman & Liston, 2020; Lutkenhoff et al., 2015).

## Discussion

### Multilevel mechanisms and pathological–functional distinctions of post-stroke hypoesthesia with anxiety/depression

Recent research exploring the relationship between hypoesthesia and emotional disorders has begun to reveal its multi-level, cross-system mechanisms. This review integrates findings from non-stroke populations regarding the comorbidity of sensory deficits with anxiety and depression and extends them to stroke patients, emphasizing that post-stroke hypoesthesia is not solely the result of localized neural damage but also stems from complex functional interactions between sensory processing and emotional regulation systems.

**Figure 2:**
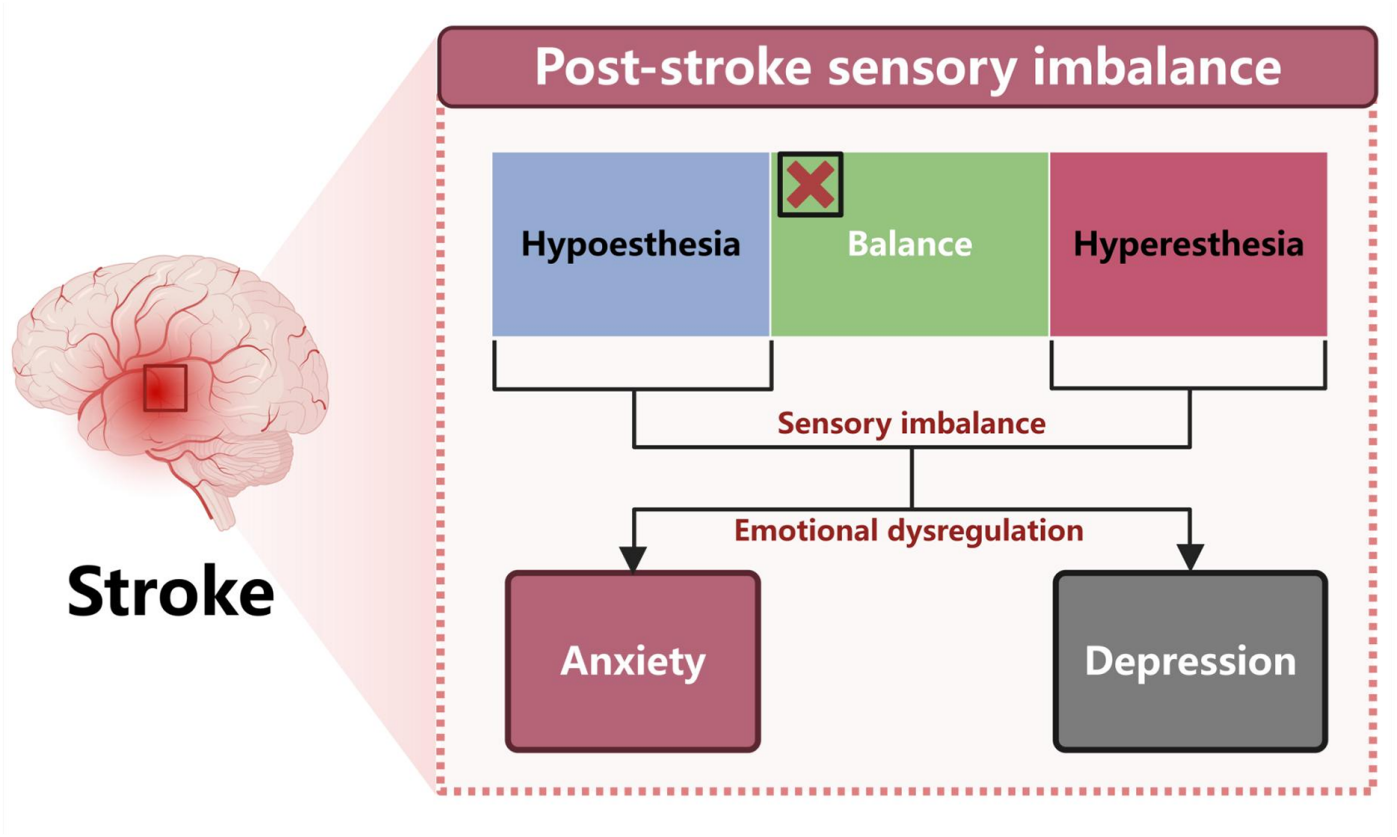
Post-stroke sensory imbalance as a trigger for emotional disorders. Stroke often leads to sensory imbalance, manifesting as either hypoesthesia or hyperesthesia. Both altered sensory states may contribute to emotional dysregulation, thereby increasing the risk of anxiety and depression.

In stroke, the sensory system is often imbalanced, presenting as hypersensitization or attenuation, each capable of inducing or worsening anxiety and depression (Fig. 2). Post-stroke hypoesthesia is rarely homogeneous; it is typically a hybrid of pathological and functional mechanisms. The pathological component reflects direct injury to the thalamus, primary and secondary somatosensory cortex, and their projections—for example, thalamo-cortical radiations—disrupting peripheral transmission or cortical responsiveness. The functional component is subtler and often extra-lesional, arising from dysregulated processing in cognitive–emotional hubs such as the insula, prefrontal cortex, and hippocampus: (1) defensive desensitization, a down-regulation of input under chronic hyperarousal or depression; (2) imbalance in semantic and attentional modulation, where disrupted allocation, context recognition, and integration render perception vague or absent; and (3) prediction-model dysfunction, where impaired input interacts with negative expectancy to produce prediction–perception mismatch and sustained interoceptive attenuation. This composite architecture helps explain the strong comorbidity with anxiety and depression.

**Figure 3:**
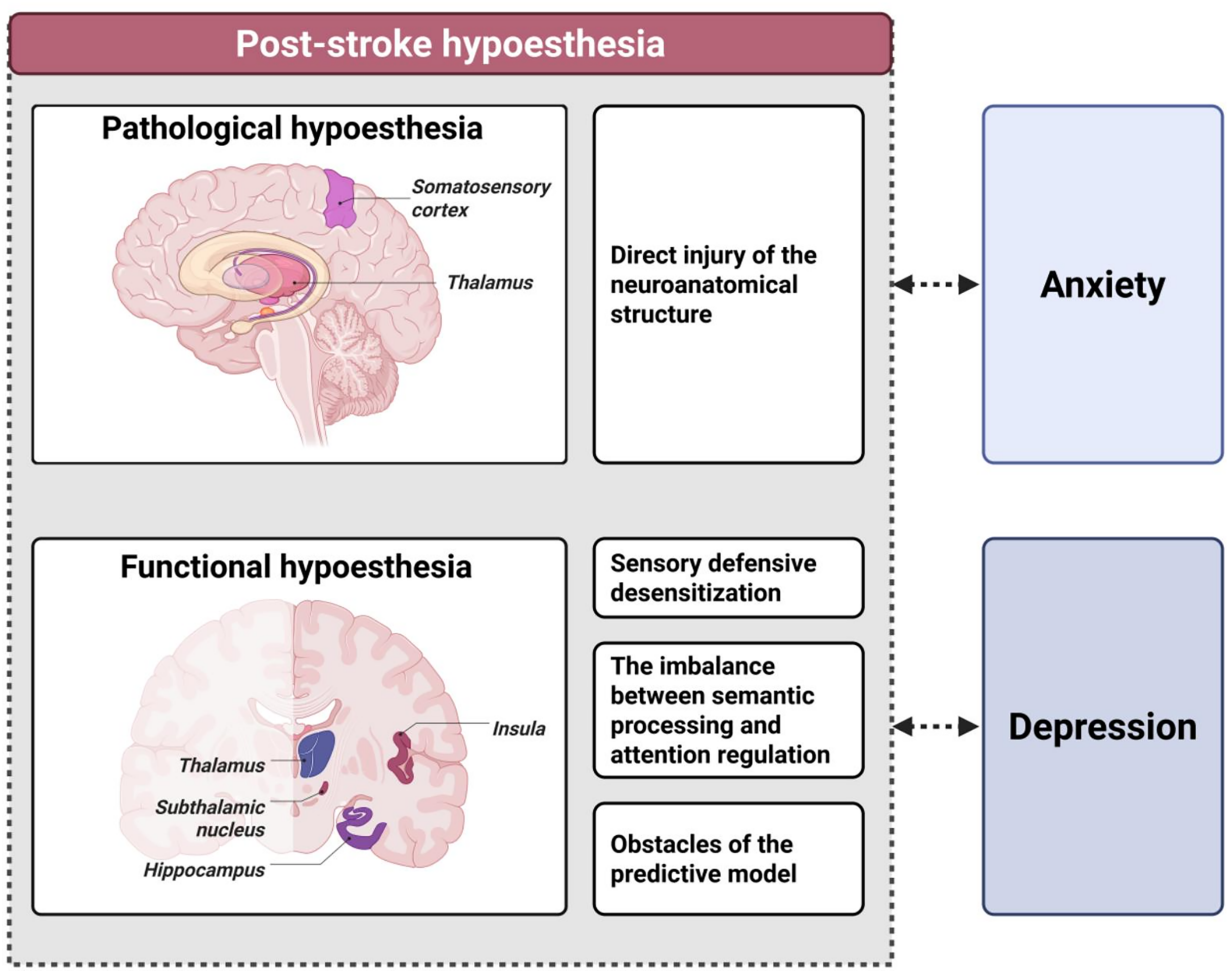
Dual mechanisms of post-stroke hypoesthesia and the emotional feedback loop. Post-stroke hypoesthesia is a mixed type of sensory impairment, comprising both pathological and functional components. Pathological hypoesthesia results from direct damage to neural structures such as the thalamus and somatosensory cortex, leading to disrupted sensory transmission. In contrast, functional hypoesthesia often occurs outside the lesion site and involves decoupling of functional connectivity in regions such as the insula, hippocampus, and subthalamic nucleus. Its mechanisms include sensory defensive desensitization, imbalance between semantic processing and attentional regulation, and disruption of predictive coding. Post-stroke hypoesthesia contributes to anxiety and depression by impairing bodily feedback, and forms a bidirectional interaction with these emotional disturbances, ultimately creating a maladaptive feedback loop.

**Figure 4:**
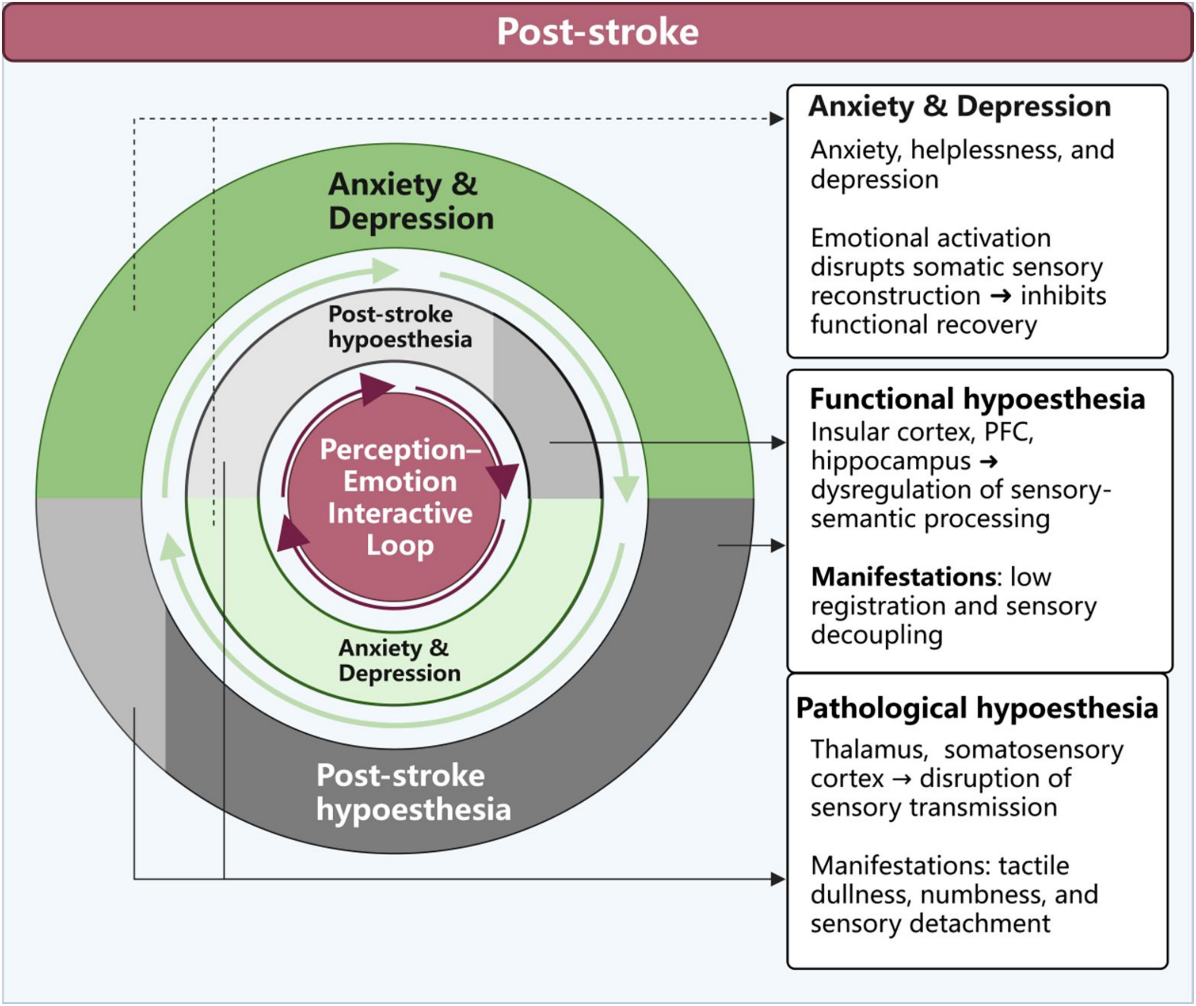
Perception–emotion interactive loop in post-stroke hypoesthesia. Post-stroke hypoesthesia involves both pathological and functional mechanisms that jointly contribute to a bidirectional cycle with anxiety and depression. Pathological hypoesthesia results from structural damage to the thalamus and somatosensory cortex, manifesting as tactile dullness, numbness, and sensory detachment. Functional hypoesthesia, on the other hand, is associated with disrupted sensory-semantic processing in the insular cortex, prefrontal cortex, and hippocampus, characterized by low registration and sensory decoupling. This sensory impairment weakens bodily feedback and triggers anxiety and depression, which in turn suppress the recovery of sensory function—forming a mutually reinforcing interaction. Within this cycle, functional hypoesthesia becomes the dominant mechanism, driving a vicious loop of “sensory degradation—emotional distress—further sensory decline,” ultimately undermining recovery motivation and the sense of self-efficacy.

This pathological × functional interplay increases vulnerability to anxiety and depression. Loss of functional perceptual feedback deprives patients of “perceptual confirmation” (“my body is recovering”), amplifying anxiety and helplessness; conversely, emotional disturbance suppresses sensory recovery. The result is a vicious cycle dominated by functional dysregulation—hypoesthesia → emotional disturbance → further hypoesthesia—in which distorted perception fuels negative expectations, further blunting perception and obstructing recovery, undermining motivation and self-efficacy (Figs. 3 and 4).

### Clinical rehabilitation implications for sensory–emotional comorbidity after stroke, limitations, and future directions

Within a functional-dysregulation–dominant framework of “hypoesthesia→emotional disorder→further hypoesthesia,” single-ended repair seldom yields stable, generalizable gains; sensory and affective targets should be addressed in parallel. In this context, hybrid hypoesthesia is both a key driver of emotional comorbidity and a basis for stratified management. The relative weight of its pathological components (lesion burden, connectivity disruption, insufficient network reorganization) and functional components (threat bias, expectancy–prediction-error imbalance, inaccurate interoception, and atypical attentional allocation) determines patients’ sensory sampling strategies, tolerance to noise, and interpretation of ambiguous signals. These, in turn, directly shape the learnability and consolidability of rehabilitation. Consequently, restoring sensory (perceptual) function should be treated as the core variable in pathway design, guiding early screening, mechanistic subtyping, and tiered intervention.

For early screening, we recommend incorporating site-specific rapid sensory tests (light touch/two-point discrimination, vibration, temperature, proprioception; key sites relevant to swallowing and gait—oropharyngeal and plantar) together with brief anxiety/depression scales at admission and discharge, and establishing sensation–emotion comorbidity risk thresholds during the subacute phase. For mechanistic subtyping, patients can be located along a pathology-dominant ↔ functional-dominant continuum: the former driven mainly by lesion load and network damage; the latter by attentional/interpretive bias and interoceptive deviation; most patients fall in between with mixed features. For tiered intervention, subtyping sets the ratio, sequence, and intensity of components: pathology-dominant profiles emphasize sensory signal amplification, channel reconstruction, and sensorimotor alignment; functional-dominant profiles emphasize threat reappraisal, attentional redistribution, and interoceptive recalibration; mixed profiles require two-track, parallel progression to avoid a “weakest-link” bottleneck that limits overall benefit.

On this basis, an integrated, sensation–emotion–centered rehabilitation pathway can be constructed. Its core is mutual calibration between sensory evidence and emotional state: when sensory discrimination improves, promptly deploy cognitive–emotional modules to reduce threat-based interpretations and avoidance; when anxiety/depression abates, immediately transfer gains to more complex sensorimotor contexts to prevent relapse. The pathway should span inpatient and home settings—precision subtyping and recovery of key nodes in hospital, high-frequency consolidation and contextual transfer at home—so as to avoid single-ended routines that focus only on sensation or only on emotion. For individualized care, an affective-profiled hypoesthesia subtype framework can be developed to target specific mechanisms in high-risk groups. In sum, breaking the vicious cycle of “hypoesthesia–anxiety/depression–hypoesthesia” hinges on acknowledging and operationalizing the dual-component nature of hybrid hypoesthesia, elevating sensory function to a core indicator for screening, subtyping, and stratification, and organizing care around co-regulation of sensation and emotion—thereby enabling earlier risk identification and interruption of the cycle, and achieving more stable functional recovery and quality-of-life gains.

Despite synthesizing substantial research from both stroke and non-stroke populations, this study has limitations. First, systematic studies on the comorbid mechanisms of sensation and emotion after stroke remain scarce, with most current literature based on cross-sectional designs, lacking longitudinal data on the evolution of sensory-emotional interactions. Second, clinical tools for objectively assessing sensory deficits are still underdeveloped, especially in domains such as pharyngeal, oral, and interoceptive pathways. Third, there is a lack of ecological data capturing the real-time dynamics of sensory responses during emotional fluctuations, making it difficult to determine whether sensory deficits are causal or maintenance factors in emotional disorders.

Future research should focus on dynamically tracking the sensation–emotion–behavior triad in stroke patients, using technologies such as neurofeedback and functional near-infrared spectroscopy (fNIRS) to monitor real-time responses of the sensory system to emotional changes. Additionally, interdisciplinary collaboration is essential to construct integrated “sensory–emotional comorbidity management pathways” within rehabilitation medicine—combining neuropsychology, speech therapy, and cognitive intervention to achieve earlier detection, more precise classification, and more comprehensive treatment.

## Conclusion

Post-stroke hypoesthesia is not only due to injured neural pathways; it also triggers, maintains and worsens anxiety and depression. Sensory function is often dysregulated as hypersensitization or attenuation, both of which intensify mood symptoms. Hypoesthesia is hybrid: a pathological route from damage to the thalamus, insula and somatosensory cortex that blunts transmission and perception, and a functional route from dysregulation in the insula, prefrontal cortex and hippocampus that skews attention and meaning-making, producing low registration and loss of feedback. Together these changes erase perceptual confirmation, promote catastrophizing and suppress motivation. Mood symptoms then further drain sensory processing and block recovery, creating a vicious cycle: hypoesthesia → anxiety or depression → more hypoesthesia. Guided by this mechanism, we propose a sensation–emotion centered rehabilitation pathway. Make sensory function the core variable for early screening with brief site-specific sensory tests and short anxiety and depression scales, for mechanistic subtyping along a continuum from pathology-dominant to functional-dominant, and for tiered intervention where the ratio, sequence and intensity match the subtype. Organizing care around joint regulation of sensation and emotion can break the cycle, enable earlier risk interception and support steadier functional recovery and better quality of life.

## Additional Information and Declarations

### Data Availability

The following information was supplied regarding data availability:

This is a literature review.
